# Conservation of Species- and Trait-Based Modeling Network Interactions in Extremely Acidic Microbial Community Assembly

**DOI:** 10.3389/fmicb.2017.01486

**Published:** 2017-08-10

**Authors:** Jialiang Kuang, Marc W. Cadotte, Yongjian Chen, Haoyue Shu, Jun Liu, Linxing Chen, Zhengshuang Hua, Wensheng Shu, Jizhong Zhou, Linan Huang

**Affiliations:** ^1^State Key Laboratory of Biocontrol, Guangdong Key Laboratory of Plant Resources and Conservation of Guangdong Higher Education Institutes, College of Ecology and Evolution, Sun Yat-sen University Guangzhou, China; ^2^Department of Microbiology and Plant Biology, Institute for Environmental Genomics, University of Oklahoma Norman, OK, United States; ^3^Department of Biological Sciences, University of Toronto-Scarborough Toronto, ON, Canada; ^4^Ecology and Evolutionary Biology, University of Toronto Toronto, ON, Canada; ^5^Earth Sciences Division, Lawrence Berkeley National Laboratory Berkeley, CA, United States; ^6^State Key Joint Laboratory of Environment Simulation and Pollution Control, School of Environment, Tsinghua University Beijing, China

**Keywords:** community assembly, interacting relationship, network conservation, acid mine drainage

## Abstract

Understanding microbial interactions is essential to decipher the mechanisms of community assembly and their effects on ecosystem functioning, however, the conservation of species- and trait-based network interactions along environmental gradient remains largely unknown. Here, by using the network-based analyses with three paralleled data sets derived from 16S rRNA gene pyrosequencing, functional microarray, and predicted metagenome, we test our hypothesis that the network interactions of traits are more conserved than those of taxonomic measures, with significantly lower variation of network characteristics along the environmental gradient in acid mine drainage. The results showed that although the overall network characteristics remained similar, the structural variation was significantly lower at trait levels. The higher conserved individual node topological properties at trait level rather than at species level indicated that the responses of diverse traits remained relatively consistent even though different species played key roles under different environmental conditions. Additionally, the randomization tests revealed that it could not reject the null hypothesis that species-based correlations were random, while the tests suggested that correlation patterns of traits were non-random. Furthermore, relationships between trait-based network characteristics and environmental properties implied that trait-based networks might be more useful in reflecting the variation of ecosystem function. Taken together, our results suggest that deterministic trait-based community assembly results in greater conservation of network interaction, which may ensure ecosystem function across environmental regimes, emphasizing the potential importance of measuring the complexity and conservation of network interaction in evaluating the ecosystem stability and functioning.

## Introduction

Fundamental mechanisms such as habitat filtering (e.g., resource limitation and abiotic stress), historical contingency (e.g., dispersal limitation, disturbance, and priority effect), and species interactions (e.g., competition and facilitation) are of particular interest in explaining the processes of community assembly (Chase, 2003; Fukami et al., 2005; Emerson and Gillespie, 2008; Fukami, 2015). These community assembly mechanisms have been the focus of tests of whether microbial biogeographic patterns conform to patterns similar to that of macroscopic animals and plants (Martiny et al., 2006). As a consequence of this review, a large number of studies have focused on the influences of contemporary environmental factors and the legacies of historical events on the spatial distribution of microbial communities. However, the importance of species interactions for microbial community assembly and how these interactions change along the environmental gradients remain largely unexplored.

Understanding community assembly of microbial communities is crucial, because the assembly mechanisms and the resulting microbial diversity patterns have important repercussions for ecosystem function. Through ecosystem-wide interaction networks, microorganisms can facilitate and accomplish diverse ecological processes and biogeochemical cycling of matter, energy, and nutrients (Raes and Bork, 2008; Faust and Raes, 2012). Microbial ecologists seek to unravel how microbes form these complex networks, how these network interactions affect community assembly processes and how the changes of co-occurrence patterns will ultimately affect the ecosystem functioning. Recent studies have reconstructed and described the co-occurrence patterns of species and/or traits in diverse natural habitats (Chaffron et al., 2010; Zhou et al., 2010, 2011; Steele et al., 2011; Barberán et al., 2012; Gilbert et al., 2012; Widder et al., 2014; Aylward et al., 2015; Ma et al., 2016), offering initial implications about whether the organization and variation of microbial co-occurrence networks along environmental gradients contribute to and affect the ecosystem function. Nevertheless, understanding the conservation of network interaction in response to the environmental changes at both species and trait levels remains largely unsolved, and which may provide important insights into the underlying mechanisms of community assembly and ecosystem stability (Cadotte et al., 2012).

**Figure 1:**
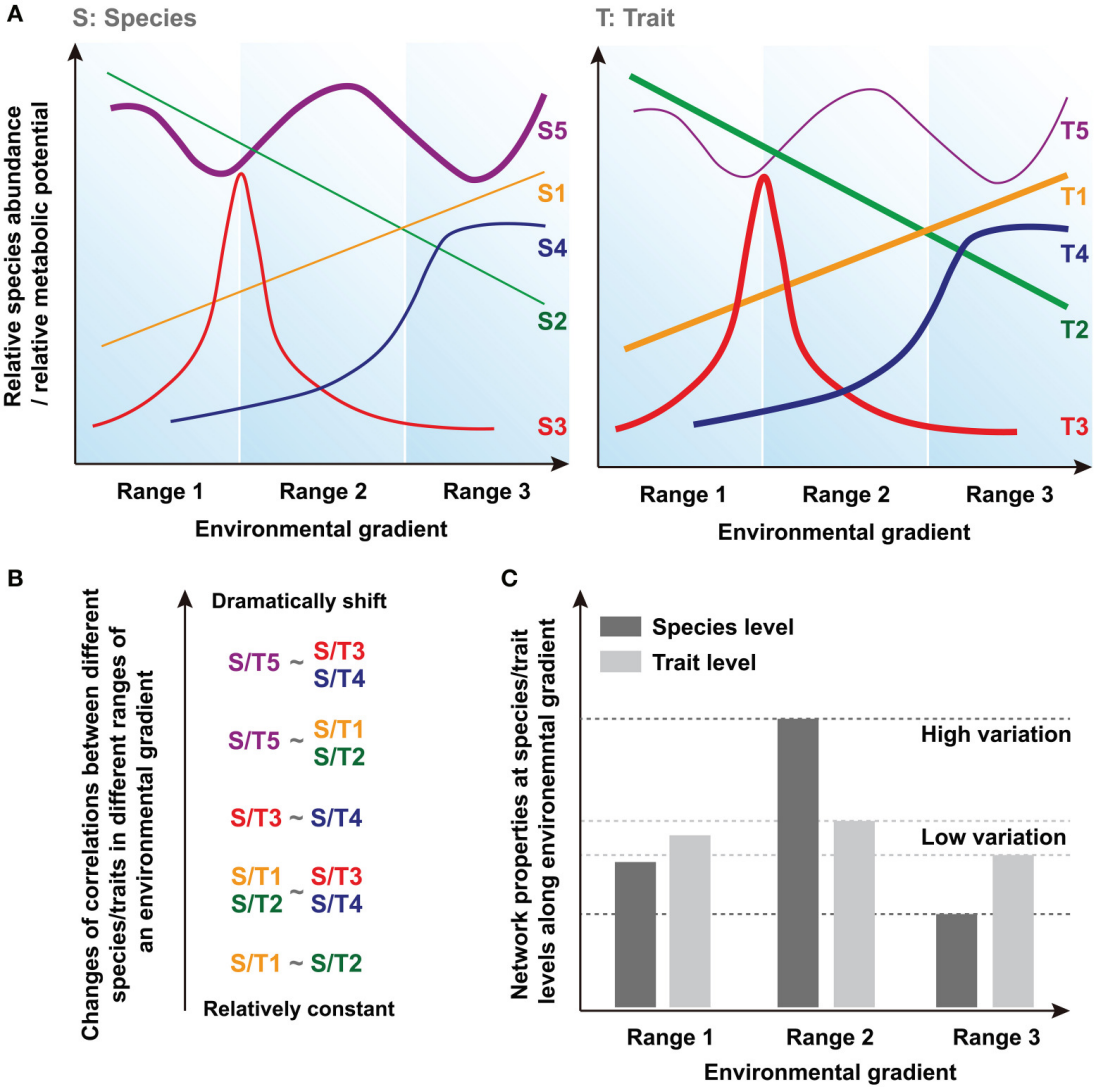
Conceptual figures showing the hypotheses regarding the differences of network conservation between species and trait levels. **(A)** The relationships between a given environmental gradient and relative species abundance or relative metabolic potential. Line thickness represents the proportion of different types of species/traits in the acid mine drainage (AMD) communities. **(B)** Changes of corretions (i.e., correlation coefficients) between different species/traits in different ranges of an environmental gradient. **(C)** Network properties and their variations at species and traits levels along environmental gradient.

Community assembly can be driven by deterministic and stochastic processes simultaneously and so both trait convergence and species divergence can be observed within the same community (Fukami et al., 2005). Indeed, increasing evidence suggests that species and traits may show distinct assembly patterns in microbial communities (Green et al., 2008; Burke et al., 2011; Raes et al., 2011; Barberán et al., 2014). Thus, species and traits may exhibit inconsistent responses to the environmental changes, and traits are expected to show greater deterministic response than that of species and converge toward a common functional structure determined by environmental conditions (Fukami et al., 2005). Basically, there are at least three types of species/traits showing different distributions, such as linear distribution (i.e., S/T1 and S/T2), normal/logarithmic distribution (i.e., with peak/maximum at optima condition, S/T3 and S/T4) and stochastic fluctuation (S/T5) along a given environmental gradient (Figure 1A). In a previous model-based prediction of the dynamics of community composition and functional attributes (Kuang et al., 2016), we show that microbial assemblages dynamics under anthropogenic disturbance, such as in acid mine drainage (AMD) sites, are better predicted using functional genes than taxonomic composition. Moreover, our previous result suggests that a higher proportion of traits are predictable under a certain condition in AMD, while most of the species tend to show less deterministic pattern of their relative abundances (proportion is represented as line thickness in Figure 1A).

Correlations based on these relative species abundances or relative metabolic potentials are widely used to uncover biologically or biochemically meaningful relationships between different species/traits across environmental gradients (Weiss et al., 2016). Generally, a positive correlation reflects mutualistic interactions or correlated environmental responses, while negative correlations suggest antagonistic relationships such as resource competition or differences in fitness optima across gradients. The correlations (i.e., correlation coefficients) between species/traits can remain relatively constant or shift dramatically in different ranges of an environmental gradient because of their distinct environmental responses (Figure 1B). Here, because higher proportion of traits, rather than species, respond more deterministic to environmental changes (i.e., most of the traits should be similar to T1~T4 as represented in Figure 1A, whereas more species are similar to Supplementary Figure S5), we hypothesize that less variation of correlation coefficients can be found at trait level. It should be noted that some species and traits will disappear entirely under a certain environmental condition. Therefore, the different patterns of correlations we supposed here are based on those species/traits that can persistently exist in a large range of environmental gradient, and the data of these species and traits are used for subsequent comparisons of network node properties.

In this study, we use network-based modeling methods to assess the ecological characteristics of these correlations, including the features of nodes (i.e., individual species/traits) and edges (i.e., different relationships between nodes) within the network (Weiss et al., 2016). Since these network interactions describe the co-occurrence of different species or traits across different samples, and not their real physical interactions directly, they are mathematically calculated based on the species- or trait-based correlations (Fuhrman and Steele, 2008; Fuhrman, 2009; Zhou et al., 2010, 2011; Barberán et al., 2012). Thus, the individual node network properties reflect its relationships with other nodes and its topological position, while the overall network structure is determined by the general characteristics of different nodes within the network. Several key network indexes of nodes and overall topology are calculated and used for the statistical tests of our hypothesis. Finally, according to our hypothesis that shown above, we suppose that the molecular ecological network (MEN) properties (i.e., network indexes) exhibit relatively higher variation at the species level because of their dramatic changes of interspecies relationships along different ranges of the environmental gradient, but remain fairly conserved at the trait level (Figure 1C).

Here, using AMD as a model system with low species richness (Denef et al., 2010), we tested the hypothesis by comprehensively comparing the species- and trait-based MENs in response to environmental changes across 40 environmental samples that were collected from diverse AMD sites across Southeast China (Kuang et al., 2013). These acidic, metal-rich and low-complexity environments exhibit a strong environmental gradient and harbor metabolically active acidophiles, and with a relatively comprehensive understanding of microbial diversity and metabolic abilities (Méndez-García et al., 2015), making them ideal systems for MENs analyses. Our results supported the hypothesis that the conservation of network interaction is significantly higher at trait level rather than at species level, and the trait-based network characteristics are more related to and affected by the environmental changes.

## Materials and methods

### Sample grouping according to the environmental pH

The geochemical properties of these 40 AMD samples are distinct along a clear gradient of environmental pH (between 1.86 and 4.10) and our previous studies (Kuang et al., 2013, 2016) demonstrated that solution pH was the most dominant factor shaping the taxonomic and functional structures in these microbial communities, revealing the distinct strategies of acidic adaptation. While other environmental properties such as Fe^2+^ and heavy metal ions were also varied at similar pH condition and influenced the microbial communities, indicating the differences of heavy metal resistance and utilization and competition of resources. In this study, we aimed to compare the structural conservation of MENs in response to the pH gradient and samples within a specific pH gradient were grouped together for the MENs construction. Specifically, we separated samples into 6 *a priori* pH groups (i.e., G1–G6) based on the solution pH, and kept a similar the sample size similar among different groups, ranging from 6 to 8 samples (the pH condition and sample size of each pH group were shown in Supplementary Table S1). To validate that whether this sample grouping can explicitly reveal the environmental gradient, we compared the differences of overall environmental properties in the entire data set and between two pH groups by permutational multivariate analysis of variance test (PERMANOVA, “adonis” function of vegan 2.3-0 in R; R Core Team, 2014; Oksanen et al., 2015) on the Euclidean distance matrixes, which are calculated by overall measured environmental properties including electrical conductivity (EC), dissolved oxygen (DO), total organic carbon (TOC), total phosphorus (P), and the concentrations of sulfate (${\text{SO}}_{4}^{2-}$), ferric (Fe^3+^), ferrous (Fe^2+^), aluminum (Al), arsenic (As), cadmium (Cd), copper (Cu), lead (Pd), and zinc (Zn) (Supplementary Figure S1A; the overall environmental properties are available in Supplementary Table S2 in Kuang et al., 2013). Additionally, principal component analysis was used to link the general pattern of overall environmental properties to distinct pH condition. Meanwhile, similar PERMANOVA and principal component analysis were also performed for different biological data sets to show their variation that explained by our sample grouping along the pH gradient (see details of the biological data sets below). Further, Bray–Curtis similarities of community composition and functional structure were calculated using species- and trait-based data sets to show their variations between samples with different values of Group Difference ranging from 0 to 5. For example, similarities of samples with Group Difference of 0 revealed variation of samples in the same pH group, while similarities of samples with Group Difference of 5 showed the beta diversities of samples between G1 and G6 (i.e., samples with most distinct pH values and environmental properties).

### Data set

In this study, relationships between different species/traits along the pH gradient were estimated by conducting pairwise Pearson correlations using three paralleled data sets (Supplementary Figure S1B). The correlations between different species were calculated based on the relative OTU (operational taxonomic unit, defined at the 97% 16S rRNA similarity level) abundances derived from 16S rRNA gene pyrosequencing data (Kuang et al., 2013). This sequencing data has been deposited in the European Nucleotide Archive database (accession no. PRJEB9908).

Meanwhile, the correlations between different traits were evaluated based on two functional data sets. One of them was the metabolic potentials (i.e., signal intensities) of diverse GeoChip probes (GCps), which covering major functional genes involved in biogeochemical processes and stress toleration and adaptation based on functional microarray (GeoChip 4.0; Tu et al., 2014; Kuang et al., 2016). This GeoChip data set is publicly available at http://ieg.ou.edu/4download/.

The other functional data set was the abundances (i.e., copy numbers) of KEGG orthologs (KOs) from different KEGG (Kyoto Encyclopedia of Genes and Genomes) categories, including metabolism, genetic information processing, environmental information processing, and cellular processes (Kanehisa and Goto, 2000). We generated this predicted metagenomic content (i.e., composition of KOs) from the 16S rRNA gene sequence data of each sample through a recently developed computational approach called PICRUSt (Langille et al., 2013), in order to provide a complementary model-based functional profiling strategy for the trait-based network construction (Supplementary Figure S1B). PICRUSt is a bioinformatics program to infer the metagenome relying on the reference genomes that pre-annotated against KEGG database and output a table of KOs abundances. The accuracy of metagenome prediction is assessed by the nearest sequenced taxon index (NSTI, the sum of phylogenetic distances for each OTU to its closely nearest related microbe) and in general decreases with increasing NSTI (Langille et al., 2013). Our samples had good NSTI values of 0.11 ± 0.07 (mean ± *SD*) according to the comparison of NSTI values across various environmental microbiomes (Langille et al., 2013), providing a reliable data set for metagenome prediction by PICRUSt.

### Network construction

In order to form reliable correlations and comparable MENs along the environmental gradient, only OTUs/GCps/KOs found in >50% of samples in each pH group were selected for subsequent analyses (Supplementary Figure S1C). Our MENs were constructed following the mathematical and bioinformatics framework developed previously (Luo et al., 2007; Zhou et al., 2010, 2011; Deng et al., 2012). Briefly, profiles of OTUs/GCps/KOs were standardized to mean value of 0 and variance value of 1. The standardized data matrixes were used for subsequent correlation analysis, and pairwise Pearson correlation coefficients were calculated to measure the similarity between OTUs/GCps/KOs across different samples in each pH group. A threshold value, which was automatically identified based on the data structure itself using the random matrix theory (RMT)-based approach, was used to perform correction on multiple pairwise correlations. The optimal threshold value was determined when the nearest neighbor spacing distribution of eigenvalues follows Poisson distribution (Luo et al., 2007). The similarity matrices were then converted into adjacency matrices using the optimal thresholds. Finally, valid correlations (i.e., co-occurrence links between different OTUs/GCps/KOs) based on the framework described above were retained for MEN construction (Supplementary Figure S1C). In total, 18 networks across 6 pH groups were constructed including 6 OTUs-MENs, 6 GCps-MENs, and 6 KOs-MENs.

### Network analyses and statistics

In this study, the coefficient of variation (CV) of the topological index, which is defined as the ratio of the standard deviation to the mean, was used to standardly assess the extent of conservation of MEN structure in response to the environmental changes (Supplementary Figure S1D). The overall network and individual node topological indexes were calculated based on the adjacency matrix (Zhou et al., 2010, 2011; Deng et al., 2012).

For the overall topology, the average geodesic distance (*avgGD*), average clustering coefficient (*avgCC*) and modularity (Clauset et al., 2004) were estimated (see the detailed definitions in the legend of Supplementary Table S2) and their CVs were subsequently calculated across the pH groups. For a given network, the *avgGD* shows how close between nodes and the *avgCC* is used to measure the extent of module structure (Zhou et al., 2010, 2011). While a module is a group of nodes that interact strongly among themselves but little with others in other modules, and the modularity reflects how a network is modular (Zhou et al., 2010, 2011). Because only a single data point of each overall network index was estimated for each MEN, thus only one CV value could be calculated across 6 pH groups and the statistical significance could not be assessed between species- and trait-based MENs. In order to perform the *t*-tests to show whether the CVs of these overall network indexes are significantly different between species- and trait-based MENs, a total of 100 random networks were generated individually for each MEN (i.e., 18 MENs in total) using the Maslov–Sneppen procedure (Maslov and Sneppen, 2002). This method keeps the numbers of nodes and links unchanged but rewires all of the links' positions based on the corresponding MEN, therefore the random networks and the original one have same size and are comparable to each other. Accordingly, the average and standard deviation for the simulated CVs were obtained based on these random networks and the statistical comparisons of the CVs between species- and trait-based MENs were applied by *t*-test using the standard deviations derived from the corresponding random networks (Zhou et al., 2010, 2011).

For the node properties, only shared nodes found in >3 networks were kept for the statistical analyses. In total, 48, 2,501, and 2,129 nodes were shared (e.g., OTUs that were found in >3 networks among 6 OTUs-MENs) among OTUs-, GCps-, and KOs-MENs, respectively. In this study, we used node connectivity (also called node degree) to describe the topological property of a node in a network. Generally, connectivity of a give node is the sum of links connecting this node with all other connected nodes, representing how strongly a node is connect to other nodes in the network (Zhou et al., 2010) and explicitly revealing a basic network feature of nodes. Since the node connectivity is dependent on the number of nodes in the network (i.e., network size) and the original value can't be directly compared between different networks, we calculated and ranked the node connectivity of each network, and normalized these ranks between 1 and 100 according to this formula:

$${\text{Rank}}_{norm}^{i}=(\;1-\frac{\text{Max}-{\text{Rank}}_{org}^{i}}{\;\text{Max}-1}\;)\times 99+1$$

where ${\text{Rank}}_{norm}^{i}\;$ is the normalized value of node *i*, ${\text{Rank}}_{org}^{i}$ is the original value of node *i* and Max gives the maximum values for the rank of node across the network. The frequency distributions of their CVs across 6 pH groups were plotted based on different data sets and the statistical significance was tested by Wilcoxon test. Additionally, these normalized ranks were compared by cross-validation under lower and higher pH conditions. Their conservation was assessed by the distances (*D-*values) between normalized ranks of nodes and diagonal line. The normalized rank of a given node lying on the diagonal line reflects that it is completely constant under lower and higher pH conditions. The *D-*values for different data sets (i.e., 48, 2,501, and 2,129 normalized ranks in OTUs, GCps, and KOs data sets, respectively) were compared and tested by ANOVA. Furthermore, the pairwise correlation coefficients of every pair of OTUs/GCps/KOs were visualized along the pH gradient. We calculated the CV of each pair along the pH gradient and compared them among different data sets using ANOVA to estimate the difference of conservation of their co-occurrence patterns. We finally assess whether these correlation patterns were non-random by performing the randomization test. Specifically, we randomized the correlation coefficients for each column (i.e., a pH group, and a total of six columns for a data set) and calculated the CV of each row (i.e., six randomized correlation coefficients) and the mean CV for all rows. We repeated this randomization 999 times to obtain 999 mean CV values and form the null distribution for each data set. We then calculated the *P*-value as the rank of the mean observed CV relative to this null distribution to estimate that whether the observed correlation patterns were significantly different from random distribution pattern.

### Random grouping of samples

To test whether the conservation of network interaction was actually related to the pH gradient, additional random grouping of our 40 AMD samples was also performed using GCps data set. All the methods for the network construction and indexes calculation that mentioned above were same except that samples were grouped along the pH gradient or randomly. Comparison of the overall topological properties of GCps-MENs were then conducted between pH-based sample grouping and random grouping.

### Association of network characteristics with environmental properties

To decipher whether the changes of network characteristics are relevant to the environmental properties, their relationships were measured by Mantel tests. A connectivity score was defined as 101 subtract the normalized rank of each node in each MEN and set as zero when the value of normalized rank was missing. Thus, this connectivity score was ranged from 0 to 100 and reflected the network characteristic of nodes. Nodes with higher connectivity score suggest that they are module hubs with strong interaction with other nodes and locate in key topological positions in the network. Meanwhile, we used node significance to identify the correlations between the nodes and the environmental properties (Zhou et al., 2010, 2011). Specifically, the node significance was calculated as the square of Spearman correlations between relative abundances of OTUs/signal intensities of GCps/abundances of KOs and every standardized environmental property (i.e., a total of 14 environmental variables as mentioned above) for all shared nodes (i.e., 48, 2,501, and 2,129 shared nodes for OTUs-, GCps-, and KOs data sets, respectively) in each MEN. Higher node significance indicates higher correlation between a given node and a certain environmental variable. Finally, the Mantel test was performed to estimate the relationship between connectivity score and node significance based on their Euclidean distance matrices across 6 pH groups for each environmental variable in different data sets. Here, we attempt to understand the importance of network structure in the ecosystem functioning that referred by the state of the environmental properties, and use this Mantel test to examine whether the change of nodes' network topology is related to and affected by environmental properties. Generally, significant relationship implies that nodes with similar network topological characteristics will have similar correlations with an environmental variable.

## Results

### Environmental properties, community composition and functional structure across different pH groups

The pH values of our samples showed a clear gradient across different pH groups (see details in Section Materials and Methods and Supplementary Table S1; a total of 6 *a priori* pH groups were defined), which was demonstrated previously as the major factor shaping the taxonomic and functional structures (Kuang et al., 2013, 2016). Consistent with this, the overall measured environmental properties were significantly different in the entire data set (PERMANOVA, *R*^2^ = 0.19, *P* < 0.05) and between groups with distinct pH values (e.g., G1 vs. G3) but remained similar between groups with narrow pH range (e.g., G1 vs. G2; Supplementary Table S1). Principal component analysis also suggested that the variation of these overall environmental properties was well explained by our sample grouping along the pH gradient (Supplementary Figure S2A). Additionally, similar trends were found for species- and trait-based data sets (Supplementary Figure S2B–D and Supplementary Table S1). Further, Bray–Curtis similarities of community composition and functional structure showed consistent patterns that both species- and trait-based similarities significantly decreased (*P* < 0.05) between samples with more distinct pH values and environmental properties (Supplementary Figure S3). These results suggested that the sample grouping used in this study could explicitly reveal the gradient of variation of environmental properties, with significant shifts in community composition and functional structure, implying that the structural variation of MENs across these pH groups may reflect their response to the changes of environmental conditions.

### The overall network structure and its variation of different MENs

The structural conservation of overall network topology was assessed using species and trait data. In total, 48, 2,501, and 2,129 nodes were shared (e.g., OTUs that were found in >3 networks among 6 OTUs-MENs) among OTUs-, GCps-, and KOs-MENs, respectively. Several overall network topological indexes including the average geodesic distance (*avgGD*), average clustering coefficient (*avgCC*) and modularity were estimated and their CVs were calculated across 6 pH groups (Supplementary Table S2). The values of the overall network topological indexes across the environmental gradient were consistent without significant differences among different data sets, however, significant differences (*P* < 0.05) were found for their CVs with higher variation in OTUs-MENs compared to those in GCps- and KOs-MENs (Supplementary Table S2). Additional comparison of the overall network topologies of GCps-MENs revealed significantly higher variations in randomly grouped data set than that were grouped based on the pH gradient (Supplementary Table S3), suggesting that the conserved trait-based network interaction was actually related to the pH gradient. Although, previous studies have focused on how environmental changes (e.g., elevated CO_2_) affect the overall network topology of species as well as functional gene network interactions in microbial communities (Zhou et al., 2010, 2011), little research has comprehensively compared their structural conservation across an environmental gradient. These results suggested that even though the overall topology remained similar, the structural variation between species- and trait-based MENs varied markedly under the same environmental gradient. This significantly lower overall structural variation at trait levels than that at species level revealed more conserved network interactions between traits during the environmental changes, and might be one of the underlying mechanisms to maintain the ecosystem stability.

### Conservation of node topological pattern among different MENs

**Figure 2:**
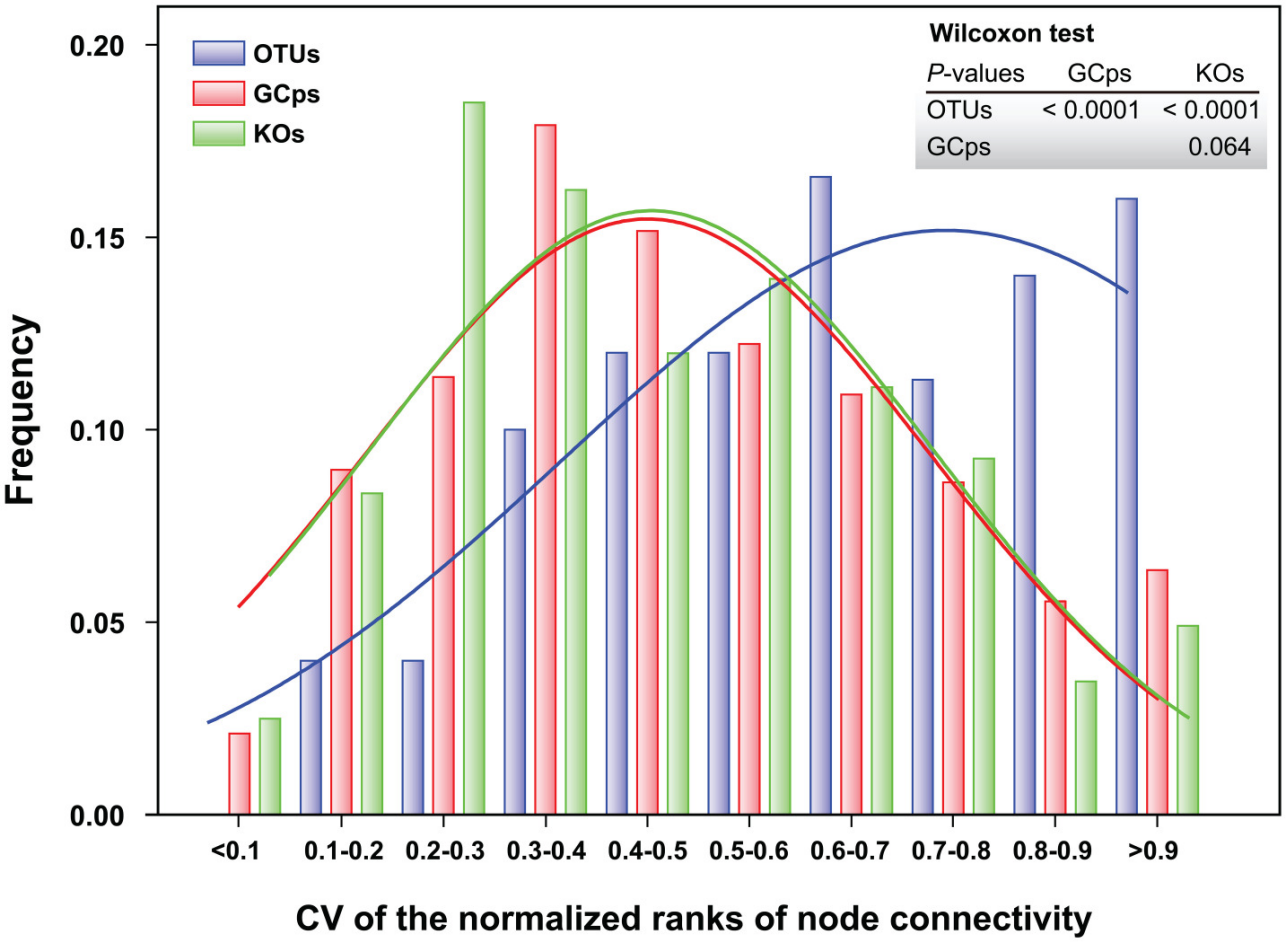
Frequency distributions of the coefficient of variation (CV) of the normalized ranks of node connectivity based on OTUs, GCps, and KOs, respectively. The difference in their distributions was tested by Wilcoxon test.

**Figure 3:**
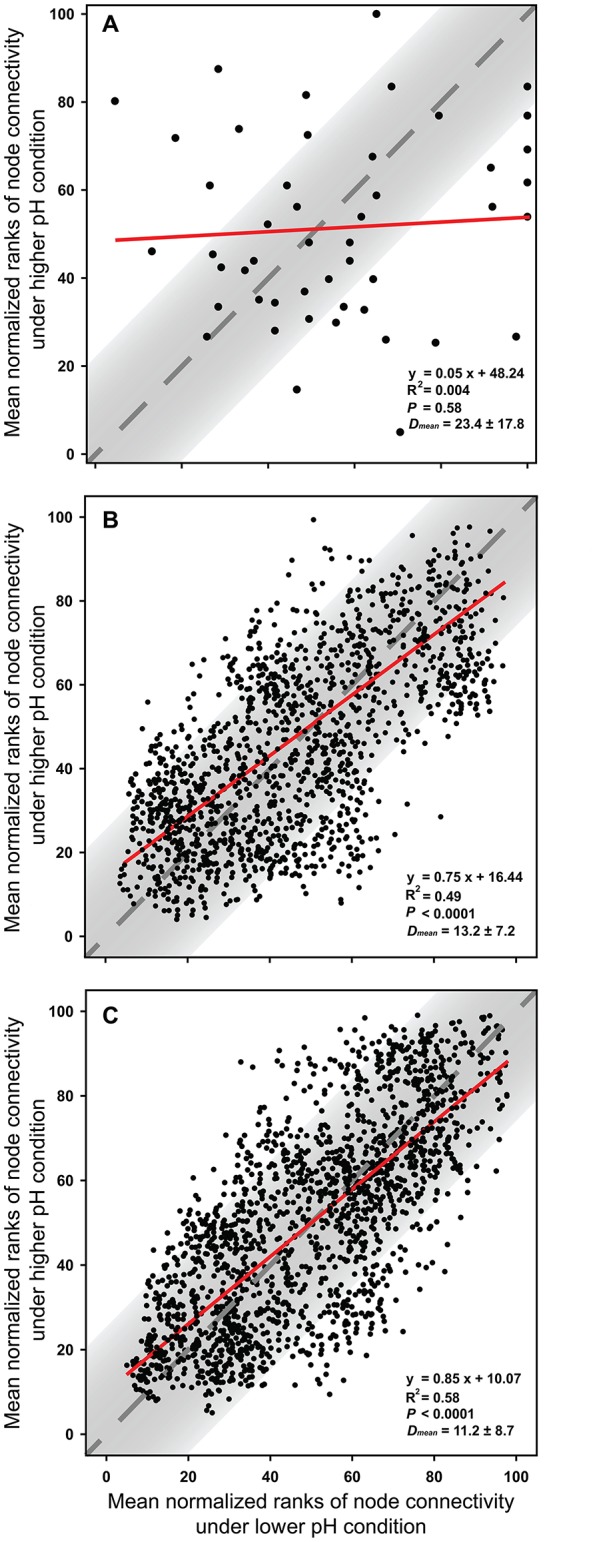
The cross-validation results of normalized rank of node connectivity based on **(A)** OTUs, **(B)** GCps, and **(C)** KOs. The mean values of normalized rank of node connectivity under lower and higher pH were calculated using the data set of environmental group G1–G3 and G4–G6, respectively. Red lines show the best-fitted linear regression models, and the normalized ranks located within gray areas (OTUs: 50%; GCps: 76%; KOs: 79%) represent <20% of the difference between lower and higher pH conditions. *D*-values are the distances between normalized ranks of nodes and diagonal line. The *D*_mean_ (mean ± *SD*) were calculated based on the normalized ranks of 48, 2,501, and 2,129 nodes in OTUs, GCps, and KOs data sets, respectively.

**Figure 4:**
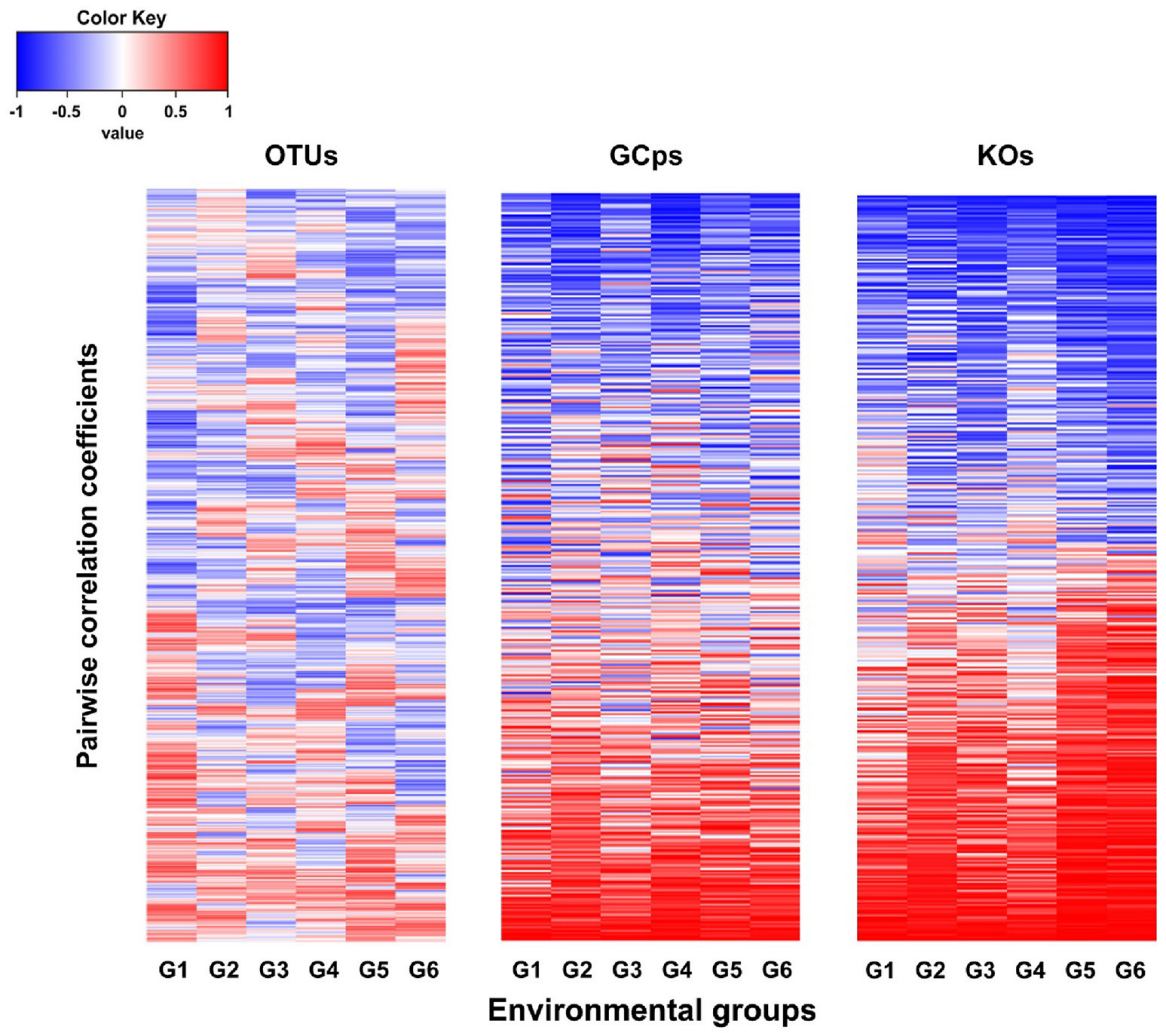
The comparison of the pairwise correlation coefficients between OTUs, GCps, and KOs across different pH groups.

The node connectivity of each MEN was ranked and normalized before statistical analyses of individual node network characteristics. The frequency distributions of the CVs clearly showed a significant difference between species- and trait-based MENs (*P* < 0.0001, Wilcoxon test; Figure 2). Specifically, the overall trend of CVs for GCps- and KOs-MENs remained similar (*P* = 0.064) but their values were significantly lower than those of OTUs-MENs. Consistently, cross-validation supported this pattern that higher percentage of nodes (GCps: 76% and KOs: 79%) revealed less variation (<20%, gray areas in Figure 3) of normalized ranks between lower and higher pH conditions with significant linear correlations (*P* < 0.0001, red lines in Figure 3) at trait levels than that at the species level (OTUs: 50%; non-significant linear correlation, *P* = 0.58). Additionally, similar results were also found that the *D*-values were significantly higher (*P* < 0.05, ANOVA) at species level compared to trait levels [*D*_OTUs_ = 23.4 ± 17.8, *D*_GCps_ = 13.2 ± 7.2 and *D*_KOs_ = 11.2 ± 8.7 (mean ± *SD*)], suggesting significantly higher variation of normalized ranks at species level (Figure 3). These results indicated that the conservation of node connectivity for trait-based MENs were higher compared to those of species-based MENs. Furthermore, the comparison of conservation of node correlations clearly showed significantly higher mean CVs of correlation coefficients between OTUs than those between GCps or KOs (*P* < 0.001, ANOVA), while no significant difference was found between GCps and KOs (*P* = 0.25; Figure 4). A randomization test revealed a non-significant *P*-value (*P* = 0.76) for the correlation patterns of OTUs and indicated that the null hypothesis that species correlations were random could not be rejected. In contrast, a significant *P*-value (*P* < 0.001) was found and suggested a non-random correlation patterns for GCps and KOs. These patterns suggested that the correlations of traits are more conserved than those of species in response to the environmental changes. In summary, our findings revealed that the topological positions of diverse traits in different MENs were relatively conserved across environmental gradients even though different species played key roles under distinct environmental conditions.

### Association of network characteristics with environmental properties

We finally examined whether the differences of network characteristics between species and trait levels could reflect their different correlations to the environmental properties. We used connectivity scores and node significance to assess the network characteristics and the correlations between nodes and environmental properties, respectively (see details in Section Materials and Methods). Mantel tests revealed stronger and more significant correlations between connectivity scores (i.e., network characteristics) and node significance (i.e., correlations between nodes and environmental properties) for all the environmental variables at trait levels than at species level (Supplementary Table S4). These results suggested that the trait-based network characteristics were more related to and affected by environmental conditions, possibly revealing more deterministic responses to the environmental changes.

## Discussion

Co-occurrence patterns not only show how particular organisms occur together under certain environmental conditions but also help to shed light on community assembly rules (Gotelli and McCabe, 2002). Our understanding of community assembly is based on the measures of the species diversity and composition, but species traits are increasingly being emphasized as important in explaining variation in community assembly and ecosystem function (Cadotte et al., 2013). Traits directly influence physiological and biochemical performance or species' fitness, and determine how species interact with one another or even the contributions of species to ecosystem function (McGill et al., 2006; Cadotte et al., 2011). By using the network-based approach, we explicitly showed distinct co-occurrence patterns between species and trait levels in an anthropogenically disturbed ecosystem. This study highlights the importance of interaction networks in understanding microbial community assembly, and more importantly, addresses how the conservation of network interaction responds to the environmental changes. Our results reveal that the conservation of trait-based network interaction stands in stark contrast to that of OTU networks, which often show high variation of network properties along environmental gradients (Widder et al., 2014) or across geographic locations (Ma et al., 2016). Further, this study clearly indicates that, although the co-occurrence (i.e., interacting relationships) at species level varied dramatically along the pH gradient, it remained strongly conserved at trait level. Such findings can be possibly explained by the fact that environmental conditions determine the available types of ecological niches that can be colonized randomly by whichever suitable species with similar traits, but that species composition within a functionally equivalent group depends on those species happen to arrive there first during the history of community assembly, that is trait-based assembly is deterministic and species-based appears much more stochastic (Fukami et al., 2005; Burke et al., 2011; Fukami, 2015). Thus, this deterministic trait-based assembly results in a more conserved network interaction among different traits along the environmental gradient.

Previous studies have reported that the metagenomic/functional composition is less variable than taxonomic composition in the human and ocean microbiomes (Human Microbiome Project Consortium, 2012; TARA Oceans Consortium, 2015). However, in our AMD samples, the variation of species- and trait-based communities can be well explained by the pH gradient (Supplementary Figure S2), and consistent patterns of their similarities were observed with significant decrease along the increase of environmental distance (Supplementary Figure S3), suggesting a stronger environmental filtering in this extreme environment. This result implied that the conserved trait-based network interaction was inherently due to the correlated or opposite environmental responses of diverse traits but not their similar functional structures. Furthermore, the test of random grouping in this study showed that the variation of the overall topology was significantly higher using the data set of random grouping than that of pH-based grouping (Supplementary Table S3). This result supports our idea that when the samples are randomly grouped, their trait distributions are no longer predictable and fluctuate stochastically among the groups. Therefore, this test, to some extent, resulted in trait distributions similar to those of relative species abundances, implying that different types of responses to pH is a key underlying mechanism explaining differences of network conservation between species and traits.

Ecosystem function broadly refers to the state of the system (e.g., stocks of materials like carbon, nitrogen, and nutrients) and the rates of processes involving fluxes of energy and matter that sustain the system, thus the environmental properties can be widely used to characterize ecosystem function (Jax, 2005). Clear evidence has revealed that changes of ecosystem functions like global CO_2_ elevation (Zhou et al., 2010, 2011) and the shifts of fluvial network hydrology (Widder et al., 2014) and soil physiochemical features (Ma et al., 2016) apparently affect the phylogenetic or functional network topology. Our observation further indicated that, compared to species-based networks, trait-based networks might be more useful in reflecting the variation of ecosystem function. This implies that although global climate change factors fundamentally impact species diversity, composition and co-occurrence patterns, higher conservation of trait-based network interaction during environmental change might result in higher ecosystem stability.

In this study, the network structural conservation and interaction pattern of the predicted metagenome were similar to those based on functional microarray data, although this functional profiling was relying on the phylogenetic data. However, the limitation of interpreting PICRUSt predictions should be considered that the detected patterns depends on the reference genomes. The relatively insufficient metagenomes in extreme habitats such as hypersaline and acidic communities may cause the prediction accuracy to appear artificially lower (Langille et al., 2013). Therefore, other community functional measures like metatranscriptome and metaproteome are needed for testing our hypothesis in further study.

Computational exploration through microbial correlation networks is considered as a necessitating technique to study the microbial communities because of their enormous complexity (Weiss et al., 2016). However, the performance and limitations are different among these computational methods, which may cause different inferring correlation networks (Weiss et al., 2016). To test whether different approaches for network construction will affect our results, SparCC, which is particularly designed to deal with compositional data (Friedman and Alm, 2012, available at https://bitbucket.org/yonatanf/sparcc), was used complementarily for our analyses of network conservation (see details in Supplementary Text). Consistent results were found by using SparCC (Supplementary Figure S4, S5 and Supplementary Table S5, S6), implying that our observed pattern of network conservation was insensitive to the approaches of network construction. Despite of this, it should be noted that our hypothesis was only tested by correlation-based methods, which may have several limitations, implying that other approaches like Bayesian network based on graphical model were still needed to further validate our findings (Friedman, 2004). Moreover, the statistical tests of overall network topologies were dependent on the random networks, which serving as a null model. However, the significant differences of the network properties between species- and trait-based networks may be an artifact due to the null model networks we used in this study (see detailed in Section Materials and Methods; Artzy-Randrup et al., 2004; Beber et al., 2012). Therefore, the interpretation of topological features between species- and trait-based networks should be addressed in future by using different null models.

In addition to the network construction, the comparison is another key challenge for interpreting the complexity and conservation among multiple biological networks. Although, we applied basic statistical analyses based on the indexes of the topological properties to assess the conservation of network characteristics, recent developed graph-theoretic algorithms provide more powerful performance to qualify the similarity and the conserved parts between networks (Pavlopoulos et al., 2011; Ali et al., 2014; Hu and Reinert, 2015; Yaveroğlu et al., 2015). Future study using these advanced methods will help us to uncover the hidden features of interaction networks, and to better evaluate the dynamics of these features during the succession of the microbial communities.

In summary, we have linked the organization and conservation of species- and traits-based network interactions to the ecological processes of community assembly in AMD system, and emphasized that in addition to the calculation of diversity indexes based on species/trait richness and abundance, measuring the complexity and conservation of their network relationships and characteristics can be considered as an alternative way to provide a further understanding of ecosystem stability and functioning (Zhou et al., 1991). Ultimately, simultaneously illustrating the changes of diversity, composition and network interaction during the succession of microbial communities using species- and trait-based data will increase our knowledge of the modeling of ecosystem dynamics, and help with the engineering and manipulation of complex microbial communities that are relevant for waste water treatment, food production and the prevention and treatment of diseases (Faust and Raes, 2012).

## Data accessibility

The 16S rRNA gene pyrosequencing data reported in this paper have been deposited in the European Nucleotide Archive database (accession no. PRJEB9908). The GeoChip data set reported in this paper is publicly available at http://ieg.ou.edu/4download/.

## Author contributions

JK, JZ, WS, and LH conceived the research. JK and MC drafted the manuscript. JK, JL, and LC carried out the field sampling. JK, MC, YC, and HS performed the analysis with advice from JL, LC, ZH, LH, JZ, and WS. JK, MC, YC, JZ, WS, and LH discussed the results and contributed to the revision of the final manuscript.

### Conflict of interest statement

The authors declare that the research was conducted in the absence of any commercial or financial relationships that could be construed as a potential conflict of interest.

## References

[B1] Ali W. Rito T. Reinert G. Sun F. Deane C. M. (2014). Alignment-free protein interaction network comparison. Bioinformatics 30, i430–i437. 10.1093/bioinformatics/btu447 25161230 PMC4147900

[B2] Artzy-Randrup Y. Fleishman S. J. Ben-Tal N. Stone L. (2004). Comment on “Network motifs: simple building blocks of complex networks” and “Superfamilies of evolved and designed networks.” Science 305:1107c. 10.1126/science.1099334 15326338

[B3] Aylward F. O. Eppley J. M. Smith J. M. Chavez F. P. Scholin C. A. DeLong E. F. (2015). Microbial community transcriptional networks are conserved in three domains at ocean basin scales. Proc. Natl. Acad. Sci. U.S.A. 112, 5443–5448. 10.1073/pnas.1502883112 25775583 PMC4418921

[B4] Barberán A. Bates S. T. Casamayor E. O. Fierer N. (2012). Using network analysis to explore co-occurrence patterns in soil microbial communities. ISME J. 6, 343–351. 10.1038/ismej.2011.119 21900968 PMC3260507

[B5] Barberán A. Ramirez K. S. Leff J. W. Bradford M. A. Wall D. H. Fierer N. (2014). Why are some microbes more ubiquitous than others? Predicting the habitat breadth of soil bacteria. Ecol. Lett. 17, 794–802. 10.1111/ele.12282 24751288

[B6] Beber M. E. Fretter C. Jain S. Sonnenschein N. Müller-Hannemann M. Hütt M. T. (2012). Artefacts in statistical analyses of network motifs: general framework and application to metabolic networks. J. R. Soc. Interface 9, 3426–3435. 10.1098/rsif.2012.0490 22896565 PMC3481585

[B7] Burke C. Steinberg P. Rusch D. Kjelleberg S. Thomas T. (2011). Bacterial community assembly based on functional genes rather than species. Proc. Natl. Acad. Sci. U.S.A. 108, 14288–14293. 10.1073/pnas.1101591108 21825123 PMC3161577

[B8] Cadotte M. W. Albert C. H. Walker S. C. (2013). The ecology of differences: assessing community assembly with trait and evolutionary distances. Ecol. Lett. 16, 1234–1244. 10.1111/ele.12161 23910526

[B9] Cadotte M. W. Carscadden K. Mirotchnick N. (2011). Beyond species: functional diversity and the maintenance of ecological processes and services. J. Appl. Ecol. 48, 1079–1087. 10.1111/j.1365-2664.2011.02048.x

[B10] Cadotte M. W. Dinnage R. Tilman D. (2012). Phylogenetic diversity promotes ecosystem stability. Ecology 93, 223–233. 10.1890/11-0426.1

[B11] Chaffron S. Rehrauer H. Pernthaler J. von Mering C. (2010). A global network of coexisting microbes from environmental and whole-genome sequence data. Genome Res. 20, 947–959. 10.1101/gr.104521.109 20458099 PMC2892096

[B12] Chase J. M. (2003). Community assembly: when should history matter? Oecologia 136, 489–498. 10.1007/s00442-003-1311-7 12836009

[B13] Clauset A. Newman M. E. Moore C. (2004). Finding community structure in very large networks. Phys. Rev. E 70:066111. 10.1103/PhysRevE.70.066111 15697438

[B14] Denef V. J. Mueller R. S. Banfield J. F. (2010). AMD biofilms: using model communities to study microbial evolution and ecological complexity in nature. ISME J. 4, 599–610. 10.1038/ismej.2009.158 20164865

[B15] Deng Y. Jiang Y. H. Yang Y. He Z. Luo F. Zhou J. (2012). Molecular ecological network analyses. BMC Bioinformatics 13:113. 10.1186/1471-2105-13-113 22646978 PMC3428680

[B16] Emerson B. C. Gillespie R. G. (2008). Phylogenetic analysis of community assembly and structure over space and time. Trends Ecol. Evol. 23, 619–630. 10.1016/j.tree.2008.07.005 18823678

[B17] Faust K. Raes J. (2012). Microbial interactions: from networks to models. Nat. Rev. Microbiol. 10, 538–550. 10.1038/nrmicro2832 22796884

[B18] Friedman J. Alm E. J. (2012). Inferring correlation networks from genomic survey data. PLoS Comput. Biol. 8:e1002687. 10.1371/journal.pcbi.1002687 23028285 PMC3447976

[B19] Friedman N. (2004). Inferring cellular networks using probabilistic graphical models. Science 303, 799–805. 10.1126/science.1094068 14764868

[B20] Fuhrman J. A. (2009). Microbial community structure and its functional implications. Nature 459, 193–199. 10.1038/nature08058 19444205

[B21] Fuhrman J. A. Steele J. A. (2008). Community structure of marine bacterioplankton: patterns, networks, and relationships to function. Aquat. Microb. Ecol. 53, 69–81. 10.3354/ame01222

[B22] Fukami T. (2015). Historical contingency in community assembly: integrating niches, species pools, and priority effects. Annu. Rev. Ecol. Evol. Syst. 46, 1–23. 10.1146/annurev-ecolsys-110411-160340

[B23] Fukami T. Martijn Bezemer T. Mortimer S. R. Putten W. H. (2005). Species divergence and trait convergence in experimental plant community assembly. Ecol. Lett. 8, 1283–1290. 10.1111/j.1461-0248.2005.00829.x

[B24] Gilbert J. A. Steele J. A. Caporaso J. G. Steinbrück L. Reeder J. Temperton B.. (2012). Defining seasonal marine microbial community dynamics. ISME J. 6, 298–308. 10.1038/ismej.2011.107 21850055 PMC3260500

[B25] Gotelli N. J. McCabe D. J. (2002). Species co-occurrence: a meta-analysis of J. M. Diamond's assembly rules model. Ecology 83, 2091–2096. 10.1890/0012-9658(2002)083[2091:SCOAMA]2.0.CO;2

[B26] Green J. L. Bohannan B. J. M. Whitaker R. J. (2008). Microbial biogeography: from taxonomy to traits. Science 320, 1039–1043. 10.1126/science.1153475 18497288

[B27] Hu J. Reinert K. (2015). LocalAli: an evolutionary-based local alignment approach to identify functionally conserved modules in multiple networks. Bioinformatics 31, 363–372. 10.1093/bioinformatics/btu652 25282642

[B28] Human Microbiome Project Consortium (2012). Structure, function and diversity of the healthy human microbiome. Nature 486, 207–214. 10.1038/nature11234 22699609 PMC3564958

[B29] Jax K. (2005). Function and “functioning” in ecology: what does it mean? Oikos 111, 641–648. 10.1111/j.1600-0706.2005.13851.x

[B30] Kanehisa M. Goto S. (2000). KEGG: kyoto encyclopedia of genes and genomes. Nucleic Acids Res. 28, 27–30. 10.1093/nar/28.1.27 10592173 PMC102409

[B31] Kuang J. L. Huang L. N. Chen L. X. Hua Z. S. Li S. J. Hu M.. (2013). Contemporary environmental variation determines microbial diversity patterns in acid mine drainage. ISME J. 7, 1038–1050. 10.1038/ismej.2012.139 23178673 PMC3635239

[B32] Kuang J. L. Huang L. N. He Z. L. Chen L. X. Hua Z. S. Jia P.. (2016). Predicting taxonomic and functional structure of microbial communities in acid mine drainage. ISME J. 10, 1527–1539. 10.1038/ismej.2015.201 26943622 PMC5029178

[B33] Langille M. G. Zaneveld J. Caporaso J. G. McDonald D. Knights D. Reyes J. A.. (2013). Predictive functional profiling of microbial communities using 16S rRNA marker gene sequences. Nat. Biotechnol. 31, 814–821. 10.1038/nbt.2676 23975157 PMC3819121

[B34] Luo F. Yang Y. Zhong J. Gao H. Khan L. Thompson D. K.. (2007). Constructing gene co-expression networks and predicting functions of unknown genes by random matrix theory. BMC Bioinformatics 8:299. 10.1186/1471-2105-8-299 17697349 PMC2212665

[B35] Ma B. Wang H. Dsouza M. Lou J. He Y. Dai Z.. (2016). Geographic patterns of co-occurrence network topological features for soil microbiota at continental scale in eastern China. ISME J. 10, 1891–1901. 10.1038/ismej.2015.261 26771927 PMC5029158

[B36] Martiny J. B. Bohannan B. J. Brown J. H. Colwell R. K. Fuhrman J. A. Green J. L.. (2006). Microbial biogeography: putting microorganisms on the map. Nat. Rev. Microbiol. 4, 102–112. 10.1038/nrmicro1341 16415926

[B37] Maslov S. Sneppen K. (2002). Specificity and stability in topology of protein networks. Science 296, 910–913. 10.1126/science.1065103 11988575

[B38] McGill B. J. Enquist B. J. Weiher E. Westoby M. (2006). Rebuilding community ecology from functional traits. Trends Ecol. Evol. 21, 178–185. 10.1016/j.tree.2006.02.002 16701083

[B39] Méndez-García C. Peláez A. I. Mesa V. Sánchez J. Golyshina O. V. Ferrer M. (2015). Microbial diversity and metabolic networks in acid mine drainage habitats. Front. Microbiol. 6:475. 10.3389/fmicb.2015.00475 26074887 PMC4448039

[B40] Oksanen J. Blanchet F. G. Kindt R. Legendre P. Minchin P. R. O'Hara R. B. (2015). Vegan: Community Ecology Package. R Package Version 2.3-5. Available online at: https://github.com/vegandevs/vegan

[B41] Pavlopoulos G. A. Secrier M. Moschopoulos C. N. Soldatos T. G. Kossida S. Aerts J.. (2011). Using graph theory to analyze biological networks. BioData Min. 4:10. 10.1186/1756-0381-4-10 21527005 PMC3101653

[B42] R Core Team (2014). R: A Language and Environment for Statistical Computing. Vienna: R Foundation for Statistical Computing Available online at: http://www.R-project.org/

[B43] Raes J. Bork P. (2008). Molecular eco-systems biology: towards an understanding of community function. Nat. Rev. Microbiol. 6, 693–699. 10.1038/nrmicro1935 18587409

[B44] Raes J. Letunic I. Yamada T. Jensen L. J. Bork P. (2011). Toward molecular trait-based ecology through integration of biogeochemical, geographical and metagenomic data. Mol. Syst. Biol. 7:473. 10.1038/msb.2011.6 21407210 PMC3094067

[B45] Steele J. A. Countway P. D. Xia L. Vigil P. D. Beman J. M. Kim D. Y.. (2011). Marine bacterial, archaeal and protistan association networks reveal ecological linkages. ISME J. 5, 1414–1425. 10.1038/ismej.2011.24 21430787 PMC3160682

[B46] TARA Oceans Consortium (2015). Structure and function of the global ocean microbiome. Science 348:1261359 10.1126/science.1261359 25999513

[B47] Tu Q. Yu H. He Z. Deng Y. Wu L. Van Nostrand J. D.. (2014). GeoChip 4: a functional gene arrays-based high throughput environmental technology for microbial community analysis. Mol. Ecol. Resour. 14, 914–928. 10.1111/1755-0998.12239 24520909

[B48] Weiss S. Van Treuren W. Lozupone C. Faust K. Friedman J. Deng Y.. (2016). Correlation detection strategies in microbial data sets vary widely in sensitivity and precision. ISME J. 10, 1669–1681. 10.1038/ismej.2015.235 26905627 PMC4918442

[B49] Widder S. Besemer K. Singer G. A. Ceola S. Bertuzzo E. Quince C.. (2014). Fluvial network organization imprints on microbial co-occurrence networks. Proc. Natl. Acad. Sci. U.S.A. 111, 12799–12804. 10.1073/pnas.1411723111 25136087 PMC4156742

[B50] Yaveroğlu Ö. N. Milenković T. Pržulj N. (2015). Proper evaluation of alignment-free network comparison methods. Bioinformatics 31, 2697–2704. 10.1093/bioinformatics/btv170 25810431 PMC4528624

[B51] Zhou J. Deng Y. Luo F. He Z. Yang Y. (2011). Phylogenetic molecular ecological network of soil microbial communities in response to elevated CO2. MBio 2, e00122–e00111. 10.1128/mBio.00122-11 21791581 PMC3143843

[B52] Zhou J. Deng Y. Luo F. He Z. Tu Q. Zhi X. (2010). Functional molecular ecological networks. MBio 1, e00169–e00110. 10.1128/mBio.00169-10 20941329 PMC2953006

[B53] Zhou J. Z. Ma S. J. Chen C. M. (1991). An index of ecosystem diversity. Ecol. Model. 59, 151–163. 10.1016/0304-3800(91)90176-2

