# Supplementary material for: Conservation of Species- and Trait-Based Modeling Network Interactions in Extremely Acidic Microbial Community Assembly
Source: Front Microbiol. 2017 Aug 10;8:1486. doi: 10.3389/fmicb.2017.01486 (PMC5554326; doi:10.3389/fmicb.2017.01486)
Supplement: Supplementary file 1 [file Table1.DOCX]

| **Supplementary Table S1 \| Summary of statistics (R^2^) from** **permutational multivariate analysis of variance test (PERMANOVA, “adonis” function of vegan in R) between two pH groups on the data sets of environmental properties, OTUs, GCps and KOs.** | | | | | | | |
| --- | --- | --- | --- | --- | --- | --- | --- |
|  | pH group | **G1** | **G2** | **G3** | **G4** | **G5** | **G6** |
|  | pH range (mean ± SD) | 1.86 - 1.98 | 2.11 - 2.39 | 2.41 - 2.57 | 2.62 - 2.75 | 2.83 - 2.95 | 3.00 - 4.10 |
|  |  | (1.92 ± 0.06) | (2.24 ± 0.12) | (2.50 ± 0.05) | (2.65 ± 0.05) | (2.89 ± 0.05) | (3.36 ± 0.43) |
| Data sets | No. of samples | 6 | 8 | 7 | 7 | 6 | 6 |
|  |  |  |  |  |  |  |  |
| **Environmental properties** | **G1** |  | 0.07 | 0.21* | 0.20* | 0.29* | 0.31* |
|  | **G2** |  |  | 0.06 | 0.07 | 0.17* | 0.18* |
|  | **G3** |  |  |  | 0.03 | 0.27* | 0.29* |
|  | **G4** |  |  |  |  | 0.15* | 0.18* |
|  | **G5** |  |  |  |  |  | 0.06 |
|  |  |  |  |  |  |  |  |
| **OTUs** | **G1** |  | 0.09 | 0.23* | 0.25* | 0.29* | 0.45* |
|  | **G2** |  |  | 0.11 | 0.13 | 0.19* | 0.26* |
|  | **G3** |  |  |  | 0.08 | 0.13* | 0.17* |
|  | **G4** |  |  |  |  | 0.13* | 0.15* |
|  | **G5** |  |  |  |  |  | 0.04 |
|  |  |  |  |  |  |  |  |
| **GCps** | **G1** |  | 0.07 | 0.18* | 0.22* | 0.33* | 0.35* |
|  | **G2** |  |  | 0.06 | 0.09 | 0.20* | 0.22* |
|  | **G3** |  |  |  | 0.11 | 0.21* | 0.25* |
|  | **G4** |  |  |  |  | 0.19* | 0.21* |
|  | **G5** |  |  |  |  |  | 0.14 |
|  |  |  |  |  |  |  |  |
| **KOs** | **G1** |  | 0.14 | 0.26* | 0.31* | 0.35* | 0.55* |
|  | **G2** |  |  | 0.06 | 0.07 | 0.17* | 0.20* |
|  | **G3** |  |  |  | 0.16 | 0.18* | 0.30* |
|  | **G4** |  |  |  |  | 0.16* | 0.21* |
|  | **G5** |  |  |  |  |  | 0.12 |
| Samples within a specific pH gradient were grouped together and compared with others using permutational multivariate analysis of variance test based on the Euclidean dissimilarity for different data sets including the standardized environmental properties, OTUs, GCps and KOs (*, *P* < 0.05) .The overall measured environmental properties included electrical conductivity (EC), dissolved oxygen (DO), total organic carbon (TOC), total phosphorus (P) and the concentrations of sulfate (SO_4_^2-^), ferric (Fe^3+^), ferrous (Fe^2+^), aluminum (Al), arsenic (As), cadmium (Cd), copper (Cu), lead (Pd) and zinc (Zn). The data sets of OTUs, GCps and KOs are the relative OTU abundances, the metabolic potentials of diverse GeoChip probes and the abundances of KEGG orthologs, respectively. Significant differences were also found for the entire data sets of environmental properties (R^2^ = 0.19, *P* *<* 0.05), OTUs (R^2^ = 0.10, *P* < 0.05), GCps (R^2^ = 0.14, *P* < 0.05) and KOs (R^2^ = 0.15, *P* < 0.05), respectively. | | | | | | | |
