# Supplementary material for: Conservation of Species- and Trait-Based Modeling Network Interactions in Extremely Acidic Microbial Community Assembly
Source: Front Microbiol. 2017 Aug 10;8:1486. doi: 10.3389/fmicb.2017.01486 (PMC5554326; doi:10.3389/fmicb.2017.01486)
Supplement: Supplementary file 2 [file Table2.DOCX]

| **Supplementary Table S2 \| Topological properties of different MENs of microbial communities under different environmental conditions using RMT-based network construction method.** | | | | | | | | | |
| --- | --- | --- | --- | --- | --- | --- | --- | --- | --- |
| **Topological properties** | **G1** | **G2** | **G3** | **G4** | **G5** | **G6** | **Mean ± SD** | **CV*^g^*** | **Simulated CV*^h^***  **Mean ± SD** |
| No. of original OTUs*^a^* | 103 | 51 | 48 | 74 | 112 | 148 | 89 ± 39 |  |  |
| Network size (*n*)*^b^* | 99 | 51 | 48 | 74 | 97 | 145 | 86 ± 36 |  |  |
| Avg connectivity (*avgK*)*^c^* | 8.93 | 31.14 | 29.00 | 45.43 | 9.07 | 24.97 | 24.76 ± 14.02 |  |  |
| Avg path length (*avgGD*)*^d^* | 3.16 | 1.78 | 1.81 | 1.78 | 3.06 | 2.03 | 2.27 ± 0.66 | 0.29***** | 0.19 ± 0.035 |
| Avg clustering coefficient (*avgCC*) *^e^* | 0.33 | 0.69 | 0.62 | 0.68 | 0.34 | 0.34 | 0.50 ± 0.18 | 0.36***** | 0.44 ± 0.110 |
| Modularity *^f^* | 0.55 | 0.27 | 0.29 | 0.27 | 0.52 | 0.21 | 0.35 ± 0.14 | 0.41***** | 0.18 ± 0.037 |
| No. of original GCps*^a^* | 6359 | 5755 | 6979 | 4870 | 7056 | 6270 | 6215 ± 817 |  |  |
| Network size (*n*) | 5111 | 3575 | 3630 | 3631 | 5363 | 5172 | 4414 ± 882 |  |  |
| Avg connectivity (*avgK*) | 16.06 | 6.25 | 3.92 | 9.68 | 14.01 | 21.37 | 11.88 ± 6.51 |  |  |
| Avg path length (*avgGD*) | 2.65 | 3.03 | 3.27 | 2.98 | 2.75 | 2.35 | 2.84 ± 0.32 | 0.11 | 0.17 ± 0.038 |
| Avg clustering coefficient (*avgCC*) | 0.54 | 0.43 | 0.45 | 0.43 | 0.45 | 0.59 | 0.48 ± 0.07 | 0.14 | 0.45 ± 0.045 |
| Modularity | 0.41 | 0.51 | 0.61 | 0.45 | 0.36 | 0.32 | 0.44 ± 0.10 | 0.23 | 0.24 ± 0.052 |
| No. of original KOs*^a^* | 4026 | 3508 | 3641 | 4002 | 4140 | 5093 | 4068 ± 558 |  |  |
| Network size (*n*) | 3474 | 3051 | 3283 | 3360 | 3725 | 4651 | 3591 ± 565 |  |  |
| Avg connectivity (*avgK*) | 27.02 | 59.08 | 58.11 | 16.39 | 23.20 | 27.44 | 35.21 ± 18.55 |  |  |
| Avg path length (*avgGD*) | 2.46 | 2.14 | 2.22 | 2.85 | 3.18 | 2.33 | 2.53 ± 0.40 | 0.16 | 0.21 ± 0.045 |
| Avg clustering coefficient (*avgCC*) | 0.41 | 0.53 | 0.47 | 0.49 | 0.44 | 0.46 | 0.47 ± 0.04 | 0.10 | 0.42 ± 0.040 |
| Modularity | 0.44 | 0.34 | 0.22 | 0.56 | 0.49 | 0.51 | 0.43 ± 0.13 | 0.30 | 0.41 ± 0.067 |
| *^a^* The number of OTUs/GCps/KOs that were originally used for network construction.  *^b^* The number of OTUs/GCps/KOs (i.e., nodes) in a network.  *^c^* Node connectivity (also called node degree) is the sum of links connecting a give node with all other connected nodes. And *avgK* is the mean of node connectivity within a network.  *^d^* *avgGD*, average geodesic distance, while $GD= \frac{1}{n(n-1)}\sum_{i\neq j} dij$, where *dij* is the shortest path between node *i* and *j*.  *^e^* $avgCC$= $\frac{\sum_{i=1}^{n} CCi}{n}$ , while $CCi= \frac{2li}{ki(ki-1)}$ , where *li* is the number of links between neighbors of node *i* and *ki* is the number of neighbors of node *i*.  *^f^* Modularity measures the extent to which nodes have more links within their own modules than expected if linkage were random. The modularity of each network is calculated as previously described (Clauset et al., 2004).  *^g^* CV, coefficient of variation.  *^h^* Simulated CV, the CV that calculated based on 100 random networks that generated by keeping the numbers of nodes and links unchanged but  rewiring all of the links based on the corresponding MENs (Maslov and Sneppen, 2002).  ***** Significant differences were found between the CVs from OTUs data set and those from GCps and KOs data sets with *P* < 0.05. | | | | | | | | | |
