# Supplementary material for: Conservation of Species- and Trait-Based Modeling Network Interactions in Extremely Acidic Microbial Community Assembly
Source: Front Microbiol. 2017 Aug 10;8:1486. doi: 10.3389/fmicb.2017.01486 (PMC5554326; doi:10.3389/fmicb.2017.01486)
Supplement: Supplementary file 3 [file Table3.DOCX]

| **Supplementary Table S3 \| Comparison of topological properties of GCps-MENs between pH-based sample grouping and random grouping using RMT-based network construction method.** | | | | | | | | | | | |
| --- | --- | --- | --- | --- | --- | --- | --- | --- | --- | --- | --- |
| **Topological properties** | **G1** | **G2** | **G3** | **G4** | **G5** | **G6** | **Mean ± SD** | **CV*^g^*** | **Simulated CV*^h^***  **Mean ± SD** | |  |
| No. of original GCps (pH-based grouping)*^a^* | 6359 | 5755 | 6979 | 4870 | 7056 | 6270 | 6215 ± 817 |  |  | |  |
| Network size (*n*) *^b^* | 5111 | 3575 | 3630 | 3631 | 5363 | 5172 | 4414 ± 882 |  |  | |  |
| Avg connectivity (*avgK*) *^c^* | 16.06 | 6.25 | 3.92 | 9.68 | 14.01 | 21.37 | 11.88 ± 6.51 |  |  | |  |
| Avg path length (*avgGD*) *^d^* | 2.65 | 3.03 | 3.27 | 2.98 | 2.75 | 2.35 | 2.84 ± 0.32 | 0.11 | 0.17 ± 0.038 | |  |
| Avg clustering coefficient (*avgCC*) *^e^* | 0.54 | 0.43 | 0.45 | 0.43 | 0.45 | 0.59 | 0.48 ± 0.07 | 0.14 | 0.45 ± 0.045 | |  |
| Modularity *^f^* | 0.41 | 0.51 | 0.61 | 0.45 | 0.36 | 0.32 | 0.44 ± 0.10 | 0.23 | 0.24 ± 0.052 | |  |
| No. of original GCps (random grouping) | 7079 | 6538 | 5334 | 5875 | 7021 | 5051 | 6145 ± 863 |  |  | |  |
| Network size (*n*) | 3920 | 2427 | 1441 | 2912 | 3581 | 2904 | 2864 ± 877 |  |  | |  |
| Avg connectivity (*avgK*) | 21.88 | 5.98 | 11.59 | 2.67 | 3.42 | 10.71 | 9.37 ± 7.14 |  |  | |  |
| Avg path length (*avgGD*) | 1.74 | 1.89 | 1.72 | 3.32 | 3.02 | 1.77 | 2.24 ± 0.73 | 0.32* | 0.18 ± 0.037 | |  |
| Avg clustering coefficient (*avgCC*) | 0.51 | 0.25 | 0.45 | 0.24 | 0.25 | 0.45 | 0.35 ± 0.12 | 0.35* | 0.40 ± 0.084 | |  |
| Modularity | 0.44 | 0.25 | 0.21 | 0.23 | 0.42 | 0.14 | 0.29 ± 0.11 | 0.40* | 0.25 ± 0.042 | |  |
| All the methods for the network construction and indexes calculation were same except that samples were grouped along the pH gradient or randomly.  *^a^* The number of GCps that were originally used for network construction.  *^b^* The number of GCps (i.e., nodes) in a network.  *^c^* Node connectivity (also called node degree) is the sum of links connecting a give node with all other connected nodes. And *avgK* is the mean of node connectivity within a network.  *^d^* *avgGD*, average geodesic distance, while $GD= \frac{1}{n(n-1)}\sum_{i\neq j} dij$, where *dij* is the shortest path between node *i* and *j*.  *^e^* $avgCC$= $\frac{\sum_{i=1}^{n} CCi}{n}$ , while $CCi= \frac{2li}{ki(ki-1)}$ , where *li* is the number of links between neighbors of node *i* and *ki* is the number of neighbors of node *i*.  *^f^* Modularity measures the extent to which nodes have more links within their own modules than expected if linkage were random. The modularity of each network is calculated as previously described (Clauset et al., 2004).  *^g^* CV, coefficient of variation.  *^h^* Simulated CV, the CV that calculated based on 100 random networks that generated by keeping the numbers of nodes and links unchanged but  rewiring all of the links based on the corresponding MENs (Maslov and Sneppen, 2002).  ***** Significant differences were found between the CVs from OTUs data set and those from GCps and KOs data sets with *P* < 0.05. | | | | | | | | | |  |  |
