# Supplementary material for: Conservation of Species- and Trait-Based Modeling Network Interactions in Extremely Acidic Microbial Community Assembly
Source: Front Microbiol. 2017 Aug 10;8:1486. doi: 10.3389/fmicb.2017.01486 (PMC5554326; doi:10.3389/fmicb.2017.01486)
Supplement: Supplementary file 4 [file Table4.DOCX]

| **Supplementary Table S4 \| Mantel tests of the relationships between the network characteristics (RMT-based network construction method) and their correlations to environmental properties.** | | | | | | |
| --- | --- | --- | --- | --- | --- | --- |
| **Environmental variables** | **OTUs** | | **GCps** | | **KOs** | |
|  | ***ρ*** | ***P*-value** | ***ρ*** | ***P*-value** | ***ρ*** | ***P*-value** |
| pH | 0.053 | 0.261 | 0.287 | 0.001 | 0.194 | 0.001 |
| DO | 0.183 | 0.001 | 0.284 | 0.001 | 0.268 | 0.001 |
| TOC | 0.082 | 0.082 | 0.172 | 0.001 | 0.154 | 0.001 |
| EC | 0.135 | 0.030 | 0.256 | 0.001 | 0.199 | 0.001 |
| SO_4_^2-^ | 0.113 | 0.028 | 0.148 | 0.001 | 0.178 | 0.001 |
| Fe^3+^ | 0.167 | 0.005 | 0.328 | 0.001 | 0.258 | 0.001 |
| Fe^2+^ | 0.129 | 0.007 | 0.261 | 0.001 | 0.183 | 0.001 |
| Al | 0.026 | 0.333 | 0.189 | 0.001 | 0.250 | 0.001 |
| Cu | 0.178 | 0.001 | 0.228 | 0.001 | 0.199 | 0.001 |
| Zn | 0.114 | 0.029 | 0.228 | 0.001 | 0.183 | 0.001 |
| As | 0.063 | 0.212 | 0.220 | 0.001 | 0.194 | 0.001 |
| Cd | 0.139 | 0.013 | 0.234 | 0.001 | 0.267 | 0.001 |
| P | 0.025 | 0.442 | 0.222 | 0.001 | 0.156 | 0.001 |
| Pb | 0.134 | 0.037 | 0.251 | 0.001 | 0.238 | 0.001 |
| To examined whether the differences of network characteristics between species and trait levels could reflect their different correlations to the environmental properties. The relationships between the network characteristics and the correlations to environmental properties were measured by Mantel tests. A connectivity score was defined as 101 subtract the normalized rank of each node and set as zero when the value of normalized rank was missing. Thus, this connectivity score was ranged from 0 to 100 and reflected the network characteristic of nodes. Nodes with higher connectivity score suggest that they are module hubs with strong interaction with other nodes and locate in a key topological position in the network. Meanwhile, we used node significance to identify the correlations between the nodes and the environmental properties. Specifically, the node significance was calculated as the square of Spearman correlations between relative abundances of OTUs / signal intensities of GCps / abundances of KOs and every standardized environmental property (i.e., a total of 14 environmental variables as mentioned above) for all shared nodes (i.e., 48, 2501 and 2129 shared nodes for OTUs-, GCps- and KOs data sets, respectively) in each MEN. Higher node significance indicates higher correlation between a given node and a certain environmental variable. Finally, the Mantel test was performed to estimate the relationship between the connectivity score and node significance based on their Euclidean distance matrixes across 6 pH groups for each environmental variable in different data sets. | | | | | | |
