# Supplementary material for: Conservation of Species- and Trait-Based Modeling Network Interactions in Extremely Acidic Microbial Community Assembly
Source: Front Microbiol. 2017 Aug 10;8:1486. doi: 10.3389/fmicb.2017.01486 (PMC5554326; doi:10.3389/fmicb.2017.01486)
Supplement: Supplementary file 7 [file Image1.PDF]

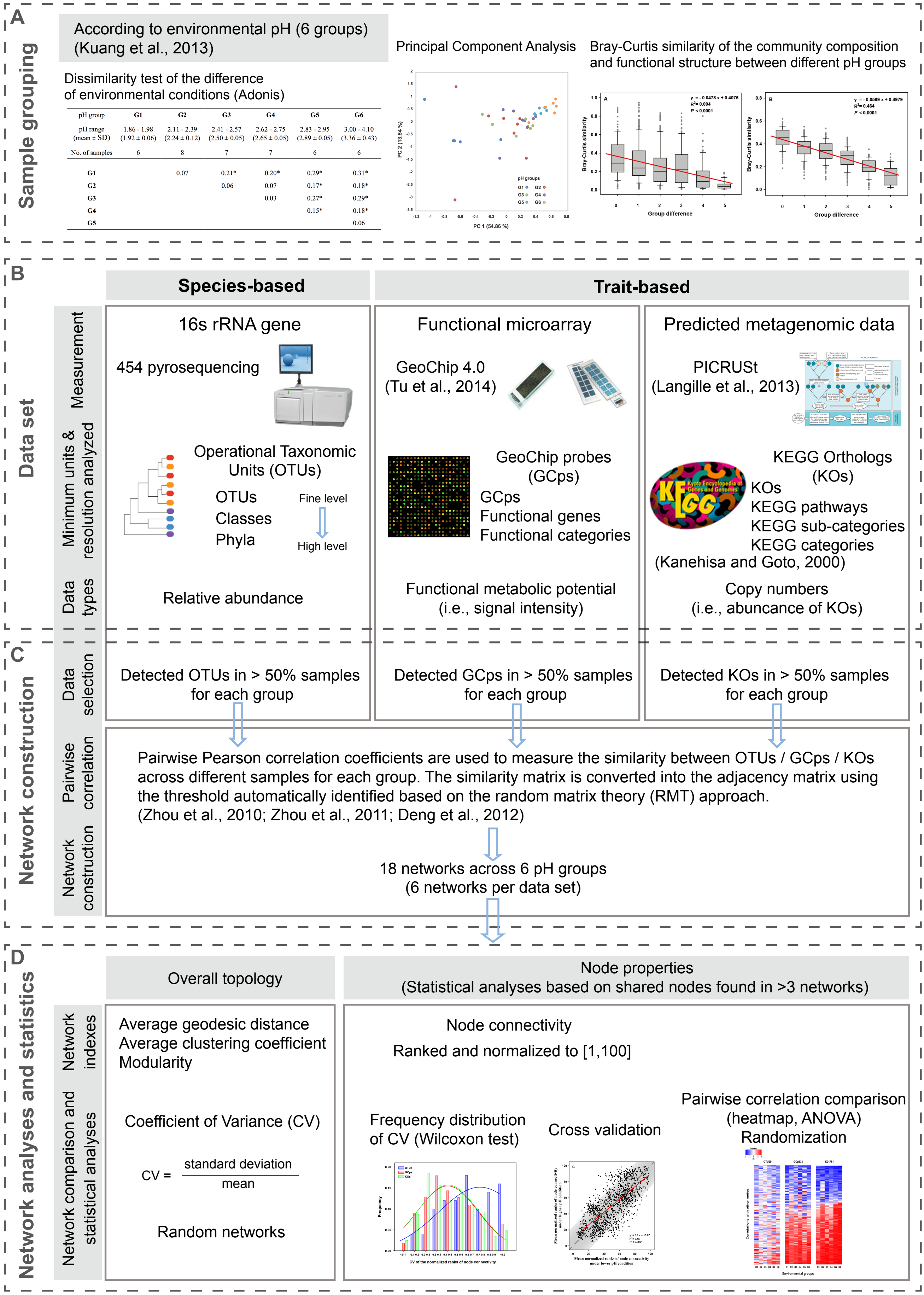

Supplementary Figure S1 | The detailed workflow and key description of the materials and methods in this study. (A) Sample grouping. (B) Data set. (C) Network construction. (D) Network analyses and statistics.
