# Supplementary material for: Conservation of Species- and Trait-Based Modeling Network Interactions in Extremely Acidic Microbial Community Assembly
Source: Front Microbiol. 2017 Aug 10;8:1486. doi: 10.3389/fmicb.2017.01486 (PMC5554326; doi:10.3389/fmicb.2017.01486)
Supplement: Supplementary file 8 [file Image2.PDF]

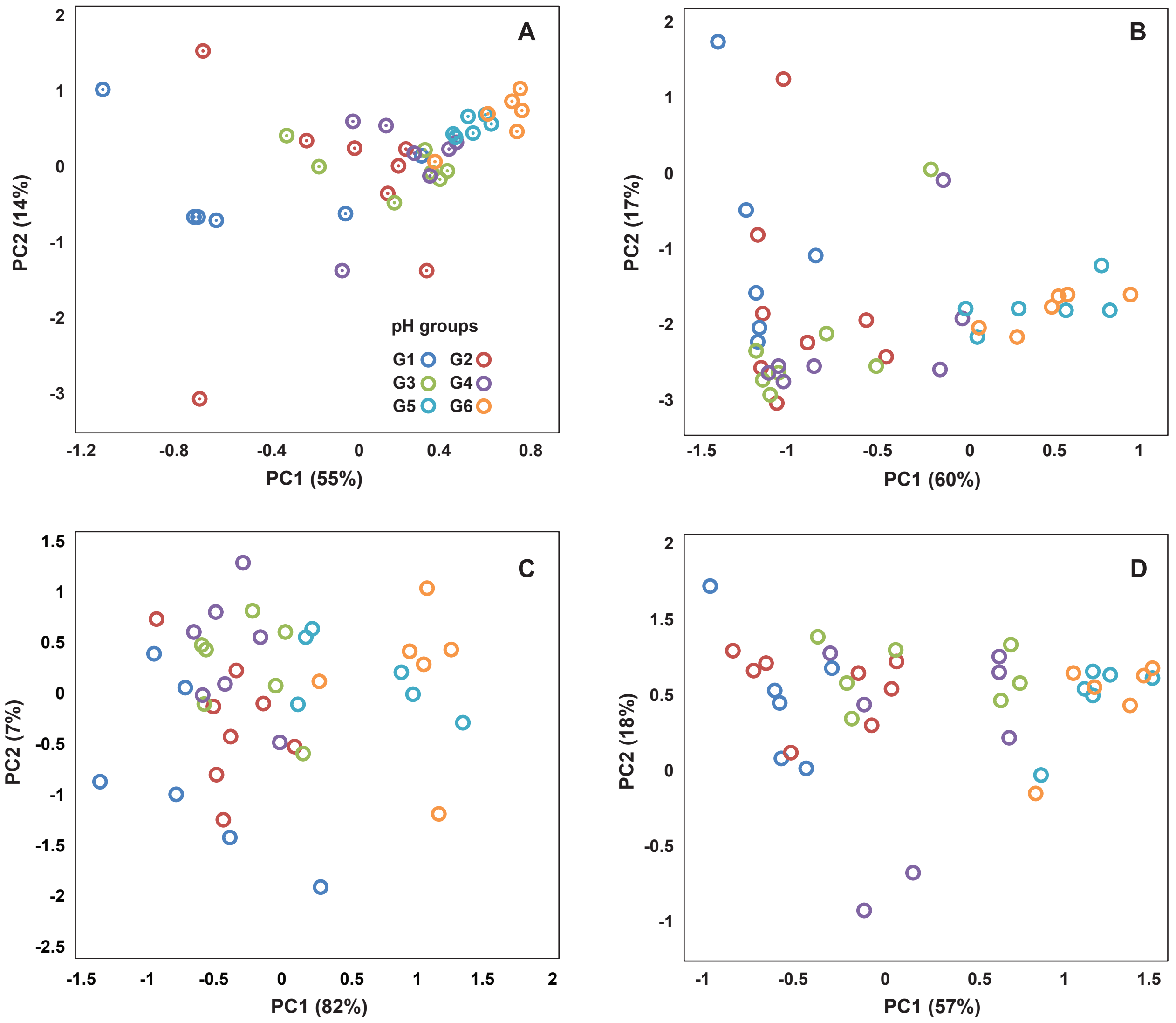

Supplementary Figure S2 | Principal component analysis linking the general pattern of (A) environmental properties, (B) relative OTUs abundances, (C) metabolic potentials and (D) abundances of KEGG orthologs to distinct pH condition.
