# Supplementary material for: Conservation of Species- and Trait-Based Modeling Network Interactions in Extremely Acidic Microbial Community Assembly
Source: Front Microbiol. 2017 Aug 10;8:1486. doi: 10.3389/fmicb.2017.01486 (PMC5554326; doi:10.3389/fmicb.2017.01486)
Supplement: Supplementary file 9 [file Image3.PDF]

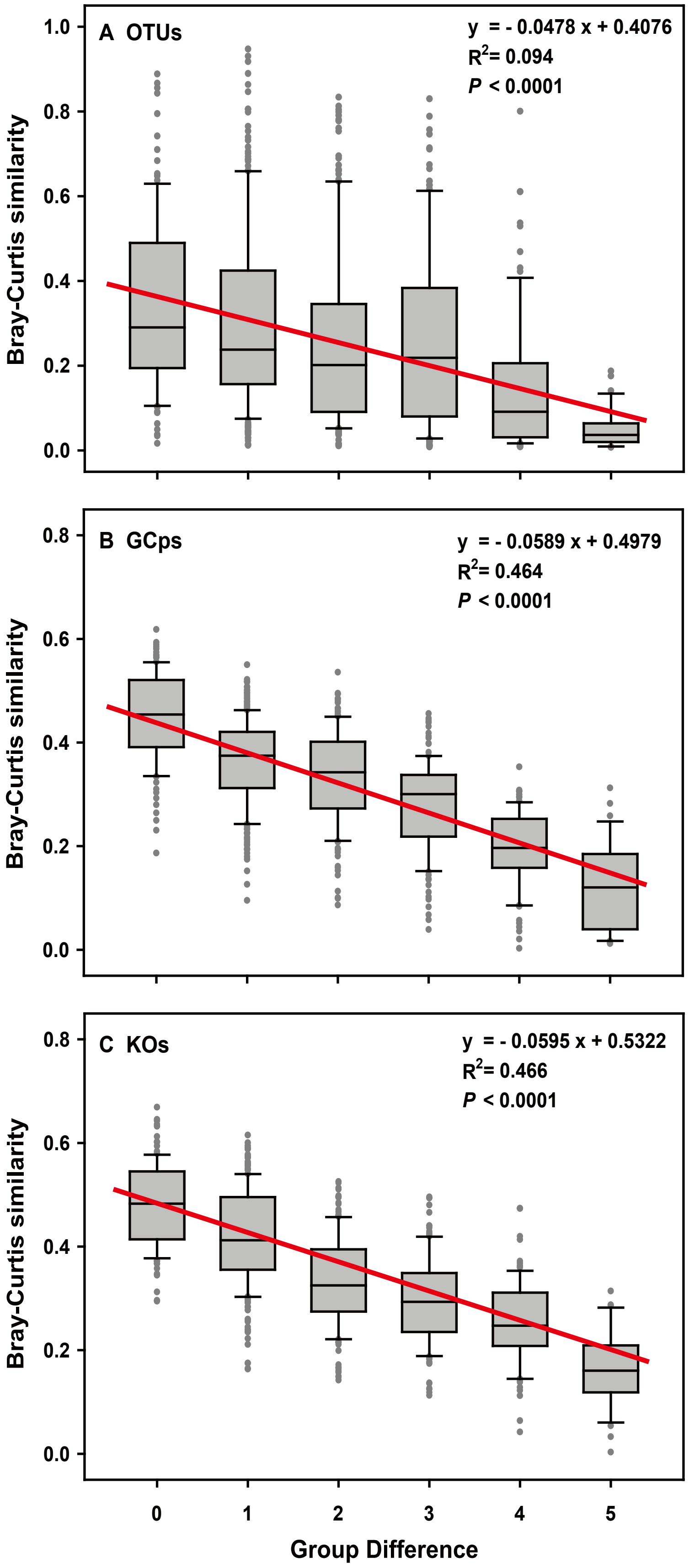

Supplementary Figure S3 | Bray-Curtis similarities of (A) community composition based on relative OTUs abundances and (B, C) functional structures based on metabolic potentials of different GCps and abundances of different KOs along the gradient of Group Difference. Similarities of samples with Group Difference of 0 revealed variation of samples in the same pH group, while similarities of samples with Group Difference of 5 showed the beta diversities of samples between G1 and G6 (i.e., samples with most distinct pH values and environmental properties).
