# Supplementary material for: Conservation of Species- and Trait-Based Modeling Network Interactions in Extremely Acidic Microbial Community Assembly
Source: Front Microbiol. 2017 Aug 10;8:1486. doi: 10.3389/fmicb.2017.01486 (PMC5554326; doi:10.3389/fmicb.2017.01486)
Supplement: Supplementary file 10 [file Image4.PDF]

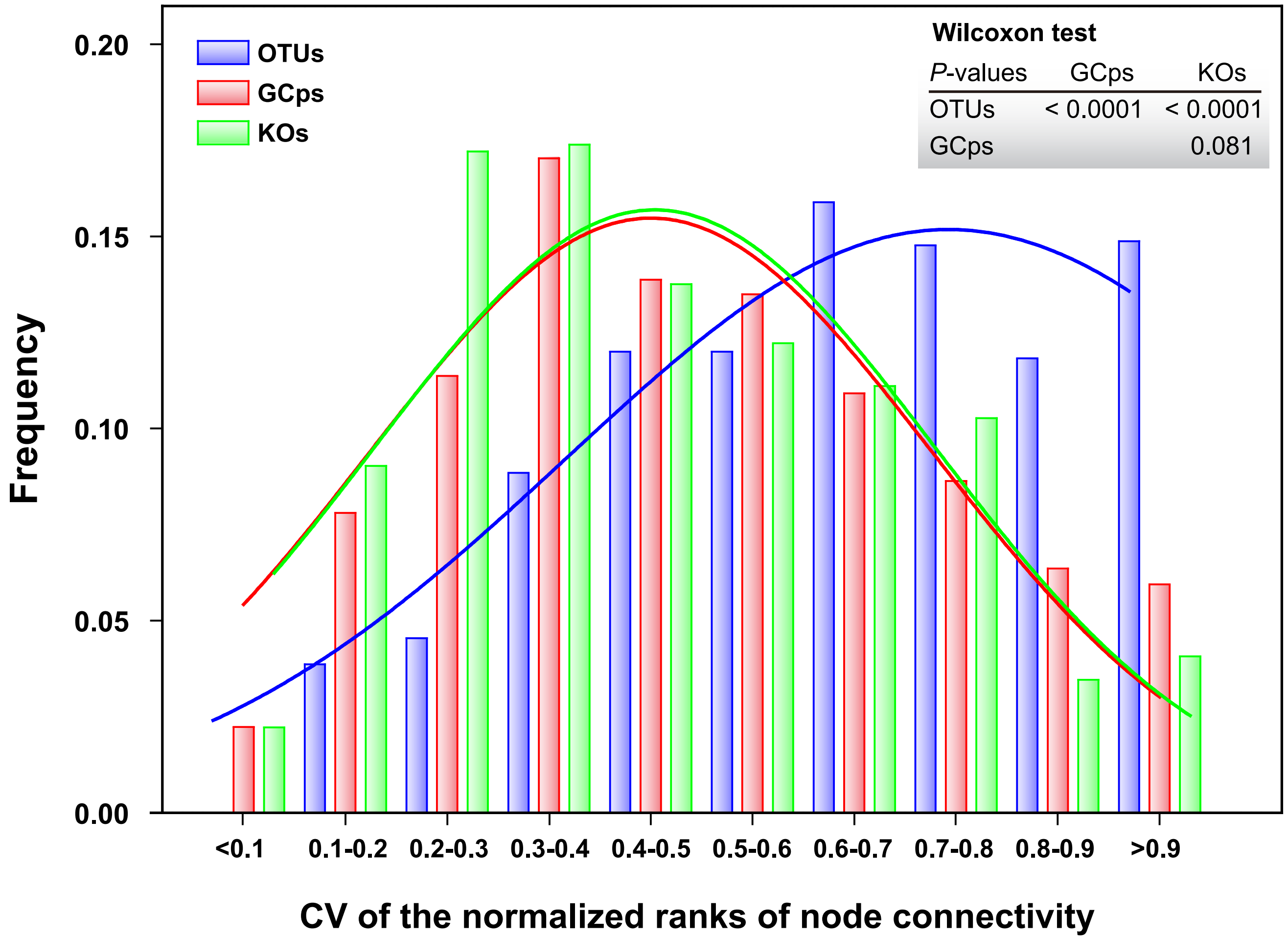

Supplementary Figure S4 | Frequency distributions of the coefficient of variation (CV) of the normalized ranks of node connectivity based on OTUs, GCps and KOs, respectively. Networks were constructed using SparCC method. The difference in their distributions was tested by Wilcoxon test.
