# Supplementary material for: Conservation of Species- and Trait-Based Modeling Network Interactions in Extremely Acidic Microbial Community Assembly
Source: Front Microbiol. 2017 Aug 10;8:1486. doi: 10.3389/fmicb.2017.01486 (PMC5554326; doi:10.3389/fmicb.2017.01486)
Supplement: Supplementary file 11 [file Image5.PDF]

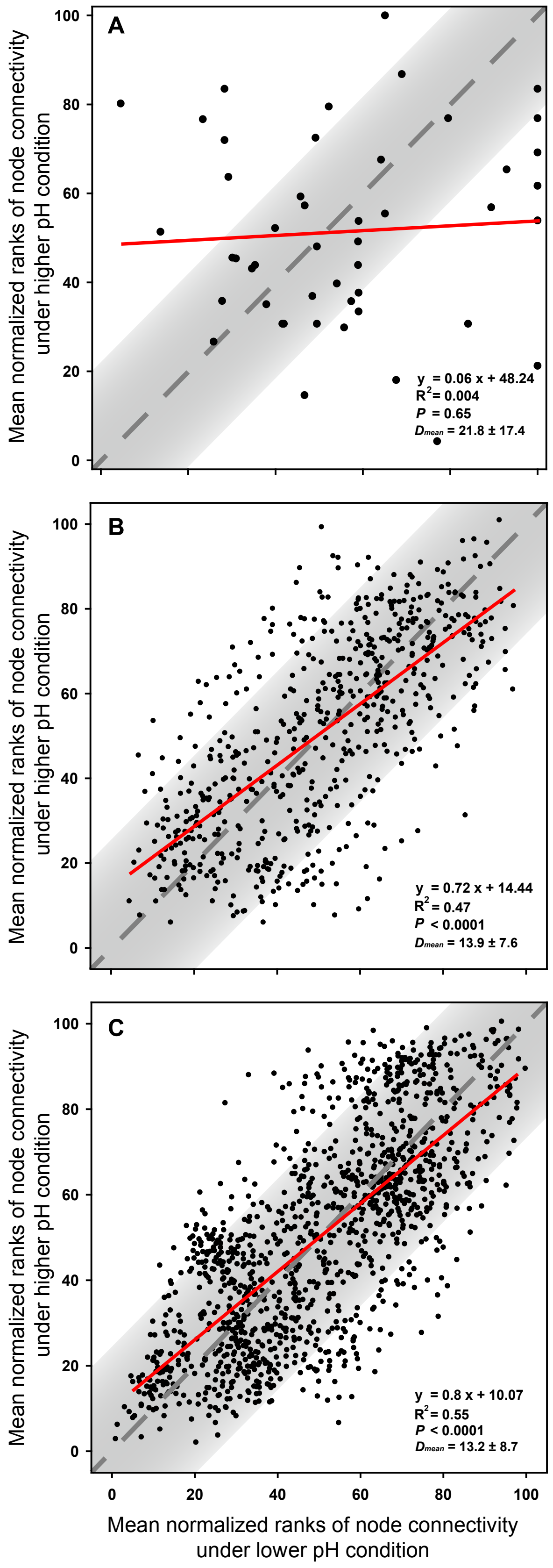

Supplementary Figure S5 | The cross-validation results of normalized rank of node connectivity based on (A) OTUs, (B) GCps and (C) KOs. Networks were constructed using SparCC method. The mean values of normalized rank of node connectivity under lower and higher pH were calculated using the data set of environmental group G1-G3 and G4-G6, respectively. Red lines show the best-fitted linear regression models, and the normalized ranks located within gray areas (OTUs: 50%; GCps: 74%; KOs: 76%) represent less than 20% of the difference between lower and higher pH conditions. *D* values are the distances between normalized ranks of nodes and diagonal line. The D_{mean} (mean ± SD) were calculated based on the normalized ranks of 50, 614 and 1243 nodes in OTUs, GCps and KOs data sets, respectively.
