# Supplementary material for: Conservation of Species- and Trait-Based Modeling Network Interactions in Extremely Acidic Microbial Community Assembly
Source: Front Microbiol. 2017 Aug 10;8:1486. doi: 10.3389/fmicb.2017.01486 (PMC5554326; doi:10.3389/fmicb.2017.01486)
Supplement: Supplementary file 12 [file Presentation1.PDF]

**Supplementary Text | Analyses and Results of Network Conservation using SparCC**

Computational exploration through microbial correlation networks is considered as a necessitating technique to study the microbial communities because of their enormous complexity (Weiss et al., 2016). However, the performance and limitations are different among these computational methods, which may cause different inferring correlation networks (Weiss et al., 2016). To test whether different approaches for network construction will affect our results, SparCC, which is particularly designed to deal with compositional data (Friedman and Alm, 2012, available at https://bitbucket.org/yonatanf/sparcc), was used complementarily for our analyses of network conservation.

Similar data sets were used as described in the main text except that only those GCps/KOs detected in all of our samples were selected for subsequent analyses, which is largely because of our limited computational power for calculating the correlations with the pseudo *P*-value via a bootstrap strategy by SparCC. The co-occurrence was considered as robust with $r > 0.6$ and $P < 0.01$ (Barberán *et al*., 2012), and these valid correlations were retained for MEN construction by the interactive platform Gephi (Bastian *et al*., 2009; Barberán *et al*., 2012). In total, 18 networks across 6 pH groups were constructed including 6 OTUs-MENs, 6 GCps-MENs and 6 KOs-MENs. Finally, we performed the statistical analyses with the same methods described in the main text.

Consistent results were found by using SparCC and RMT-based network construction methods. Briefly, for overall network structure, a total of 50, 614 and 1243 nodes were shared in > 3 networks among OTUs-, GCps- and KOs-MENs, respectively. Although the overall network topological indexes including the average geodesic distance (*avgGD*), average clustering coefficient (*avgCC*) and modularity remained consistent, their CVs were different with significantly higher ($P < 0.05$) values at species level (**Supplementary Table S5**).

For individual node topological pattern, the node connectivity of each MEN was ranked and normalized before subsequent statistical analyses. The frequency distribution of the CVs in OTUs-MENs was significantly different (*P* < 0.0001, Wilcoxon test) to those in GCps-/KOs-MENs with significantly higher CV values (*P* < 0.05) (**Supplementary Figure S4**). The cross-validation revealed that higher percentage of nodes (GCps: 74% and KOs: 76%) revealed less variation (< 20%, gray areas in **Supplementary Figure S5**) of normalized ranks between lower and higher pH conditions with significant linear correlations (*P* < 0.0001, red lines in **Supplementary Figure S5**) at trait levels than that at the species level (OTUs: 50%; non-significant linear correlation, *P* = 0.65). Meanwhile, the *D* values were significant higher (*P* < 0.05, ANOVA) at species level compared to trait levels ($D_{OTUs}$ = 21.8 ± 17.4, $D_{GCps}$ = 13.9 ± 7.6 and $D_{KOs}$ = 13.2 ± 8.7 (mean ± SD)), suggesting significantly higher variation of normalized ranks at species level (**Supplementary Figure S5**).

For the relationships between the network characteristics and their correlations to the environmental properties, our Mantel tests revealed stronger and more significant correlations between connectivity scores (i.e., network characteristics) and node significance (i.e., correlations between nodes and environmental properties) for all the environmental variables at trait levels than at species level (**Supplementary Table S6**).

In summary, theses consistent results using different network construction technologies of SparCC and RMT-based methods implied that our observed pattern of network conservation was insensitive to the approaches of network construction.

## References

Barberán, A., Bates, S.T., Casamayor, E.O., and Fierer, N. (2012). Using network analysis to explore co-occurrence patterns in soil microbial communities. *ISME J.* 6, 343-351. doi: 10.1038/ismej.2011.119

Bastian, M., Heymann, S., Jacomy, M. (2009). Gephi: an open source software for exploring and manipulating networks. In International AAAI conference on weblogs and social media, San Jose, California.

Friedman, J., and Alm, E.J. (2012). Inferring correlation networks from genomic survey data. *PLoS Comput. Biol.* 8, e1002687. doi: 10.1371/journal.pcbi.1002687

Weiss, S., Van Treuren, W., Lozupone, C., Faust, K., Friedman, J., Deng, Y., et al. (2016). Correlation detection strategies in microbial data sets vary widely in sensitivity and precision. *ISME J.* 10, 1669-1681. doi:10.1038/ismej.2015.235
